# Modes of Metabolic Compensation during Mitochondrial Disease Using the Drosophila Model of ATP6 Dysfunction

**DOI:** 10.1371/journal.pone.0025823

**Published:** 2011-10-03

**Authors:** Alicia M. Celotto, Wai Kan Chiu, Wayne Van Voorhies, Michael J. Palladino

**Affiliations:** 1 Department of Pharmacology and Chemical Biology, University of Pittsburgh School of Medicine, Pittsburgh, Pennsylvania, United States of America; 2 Pittsburgh Institute for Neurodegenerative Diseases, University of Pittsburgh School of Medicine, Pennsylvania, United States of America; 3 Molecular Biology Program, New Mexico State University, Las Cruces, New Mexico, United States of America; Yale School of Medicine, United States of America

## Abstract

Numerous mitochondrial DNA mutations cause mitochondrial encephalomyopathy: a collection of related diseases for which there exists no effective treatment. Mitochondrial encephalomyopathies are complex multisystem diseases that exhibit a relentless progression of severity, making them both difficult to treat and study. The pathogenic and compensatory metabolic changes that are associated with chronic mitochondrial dysfunction are not well understood. The *Drosophila ATP6^1^* mutant models human mitochondrial encephalomyopathy and allows the study of metabolic changes and compensation that occur throughout the lifetime of an affected animal. *ATP6^1^*animals have a nearly complete loss of ATP synthase activity and an *acute* bioenergetic deficit when they are asymptomatic, but surprisingly we discovered no *chronic* bioenergetic deficit in these animals during their symptomatic period. Our data demonstrate dynamic metabolic compensatory mechanisms that sustain normal energy availability and activity despite chronic mitochondrial complex V dysfunction resulting from an endogenous mutation in the mitochondrial DNA. *ATP6^1^*animals compensate for their loss of oxidative phosphorylation through increases in glycolytic flux, ketogenesis and Kreb's cycle activity early during pathogenesis. However, succinate dehydrogenase activity is reduced and mitochondrial supercomplex formation is severely disrupted contributing to the pathogenesis seen in *ATP6^1^* animals. These studies demonstrate the dynamic nature of metabolic compensatory mechanisms and emphasize the need for time course studies in tractable animal systems to elucidate disease pathogenesis and novel therapeutic avenues.

## Introduction

Normal metabolic pathways in animals have been elucidated and extensively studied for decades; however, the response of each pathway to the loss or disturbance of another is poorly understood. The eukaryotic cell and its mitochondria have evolved different methods of energy production from the catabolism of most food products. However, there are many human diseases that disrupt, typically via genetic hypomorphic mutations, one of these pathways. Such heritable diseases known collectively as inborn errors of metabolism do not immediately cause death; they do, however, lead to poorly understood diseases including enzymopathies and mitochondrial encephalomyopathies. Metabolic pathways are complex networks, therefore a single perturbation resulting from a single gene mutation can lead to dramatic changes in an animal's ability to maintain its normal physiological functions as well as homeostatic impairment that affects its ability to cope with environmental stresses [1].

Our current understanding of mitochondrial disease has been facilitated by the study of cellular cybrids bearing human disease mutations. However, such systems have not yielded a clear picture of the bioenergetics and compensatory mechanisms that exist within the tissues of an intact animal with mitochondrial disease. Thus, no comprehensible understanding of the associated pathogenesis has resulted, demonstrating the inherent difficulty in using cellular models to study multisystem diseases [2], [3]. Additionally, these diseases typically exhibit an asymptomatic period varying from days to decades, onset, and a stereotyped progression of the disease making them difficult to model in cellular systems. Little is known about disease pathogenesis in an intact animal with functional neurons and muscle fibers that can be examined over the life of the animal. Thus, it is essential to study the progressive nature and tissue-specific attributes of these diseases with the goal of identifying endogenous compensatory mechanisms that might be exploited as therapeutic avenues.

Here we utilize a novel, well-characterized, endogenous mitochondrial mutation in the *ATP6* gene (NC_001709.1) of *Drosophila melanogaster* with a nearly complete loss of ATP synthase activity [4]. These *Drosophila* mutants have a missense mutation in *ATP6* (G to A transition resulting in a glycine to glutamate change at position 116 in the protein), the mitochondrial gene encoding subunit 6 of the F_1_F_o_-ATP synthase (complex V of the respiratory chain) [4], [5], [6], [7], [8]. ATP6 allows for the hydrogen ion translocation required for the rotation of the F_o_ motor and the production of ATP from ADP [9]. *Drosophila ATP6^1^* mutants model human mitochondrial encephalomyopathy and demonstrate phenotypes associated with degenerative disease, including: reduced longevity, mitochondrial pathology, progressive neural dysfunction, tissue degeneration and locomotor impairment [4]. In humans, 8 missense and two frame shift mutations lead to ATP6 impairment and are known to cause the related mitochondrial disorders: maternally inherited Leigh's syndrome (MILS), neuropathy, ataxia, and retinitis pigmentosa (NARP), and familial bilateral striatal necrosis (FBSN) [10], [11], [12], [13], [14], [15], [16], [17], [18]. These diseases are characterized by reduced longevity, progressive neuromuscular impairment, seizures, myodegeneration and a range of devastating complications resulting from renal, cardiac, endocrine and hepatic system dysfunction [19], [20], [21], [22], [23], [24], [25], [26], [27], [28]. The diversity of symptoms and phenotypes associated with ATP6 dysfunction in humans and flies likely reflects this protein's important and highly conserved role in cellular bioenergetics. The pathological basis of diseases associated with ATP6 impairment in humans is not understood but it has been hypothesized that there may be uncoupling of complex V resulting in bioenergetic impairment and oxidative stress owing to respiratory chain dysfunction [29], [30].

Our results demonstrate that there are dynamic adjustments made within many of the metabolic pathways over the lifetime of animals with ATP6 dysfunction, which allow them to maintain a normal level of energy, despite the severe reduction in ATP production through oxidative phosphorylation (OXPHOS). Glycolysis and ketogenesis compensate for the OXPHOS defect earlier in life. We also demonstrate that a loss in mitochondrial supercomplex formation and complex II activity are associated with pathogenesis. These data demonstrate that mitochondrial encephalomyopathies results in dynamic metabolic compensation and that disease pathogenesis does not result from a loss of energy and involves a cascade of events broadly affecting metabolic and mitochondrial function.

## Results

### *ATP6^1^* survival and behavioral changes

**Figure 1:**
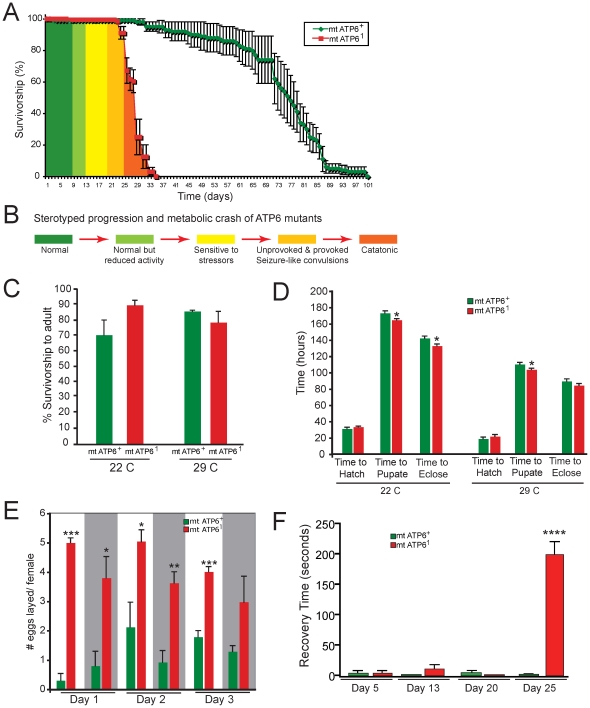
Phenotypic progression of *ATP6^1^* animals. A) Longevity curve showing wildtype (green) and *ATP6^1^* (red) demonstrating an ∼40% reduction in lifespan. Green, yellow and orange shading describes the change in animal behavior over *ATP6^1^* lifespan. Life spans are based upon 80 total animals per genotype. Error is S.E.M. Statistical analysis is log-rank. B) Summary of phenotypic progression of *ATP6^1^* animals. C) Graph showing normal developmental survival rate for *ATP6^1^* compared to wildtype at 22°C and 29°C. N = 510 *ATP6^1^* at 22°C, N = 173 wildtype at 22°C, N = 696 *ATP6^1^* at 29°C, N = 586 wildtype at 29°C. Error is S.E.M. Statistical analysis is student's t-test. D) *ATP6^1^* mutants develop significantly faster than controls at 22°C (to eclosion) and at 29°C (to pupation). N = 30 both genotypes both temperatures. Error is S.E.M. Statistical analysis is student's t-test. E) *ATP6^1^* animals exhibit a higher fecundity early in adulthood. Gray represents dark intervals of a 12∶12 light dark regime. N = 462 *ATP6^1^* , N = 139 wildtype. Error is S.E.M. Statistical analysis is student's t-test. F) Strobe lighting induces seizure-like convulsions followed by paralysis only in aged *ATP6^1^* animals. Young mutants and controls did not exhibit convulsions or paralysis. *ATP6^1^* day 5 N = 17; day 13 N = 19; day 20 N = 13; day 25 N = 23. Wildtype day 5 N = 14; day 13 N = 23; day 20 N = 13; day 25 N = 25. Error is S.E.M. Statistical analysis at day 25 is a two-tailed Mann-Whitney U test. Also see S1A–C and S2A–C.

*ATP6^1^ Drosophila* mutants exhibit a stereotyped phenotypic progression that is analogous to the symptomatic progression reported for many human mitochondrial disease patients [5], [14]. *ATP6^1^* mutant flies demonstrate stress sensitivity, shortened lifespan, muscle degeneration and abnormal mitochondrial morphology [4]. *ATP6^1^* animals eclose looking and acting completely normal and are morphologically and behaviorally indistinguishable from wildtype animals. *ATP6^1^* animals exhibit a stereotypical progression of disease following onset (∼day 8) when the animals begin to have reduced locomotor activity (Figure 1A and 1B). By day 13, *ATP6^1^* animals are sensitive to mechanical stress resulting in paralysis, suggesting neuromuscular impairment. At ∼day 20, *ATP6^1^* animals can be observed having sporadic and unprovoked seizure-like activity. Late in pathogenesis *ATP6^1^* phenotypes continue to worsen until their premature death (Figure 1B).

We discovered that *ATP6^1^* animals have a similar developmental survival rate as wildtype animals whether they are raised at 22°C or 29°C (Figure 1C). Interestingly, *ATP6^1^* animals show a modest but significant shortening in the time of development during the larval and pupal stages at 22°C and during the larval stage at 29°C (Figure 1D). Additionally, *ATP6^1^* females lay significantly more eggs than wildtype animals during their first week of life (Figure 1E). These data demonstrate that the altered physiology of *ATP6^1^* animals results in an accelerated development and increase in female fecundity. Such effects could cause increase utilization of energy early in life to ensure survival despite the dramatically altered physiology.

To examine the seizure behavior we asked whether sensory hyperstimulation, such as a strobe light, could elicit seizure behavior in *ATP6^1^* flies. Video analysis of locomotor function prior to, during, and following 1450 fpm (flashes per minute) strobe lighting (20 seconds) was used to examine the ability to induce seizure behavior by sensory hyperstimulation alone. Although strobe lighting did not affect the locomotion of wildtype flies (Video S1, Video S2, Video S3), *ATP6^1^* animals exhibited convulsive behavior with a high penetrance both during and after the strobe light (Video S4, Video S5, Video S6). Surprisingly, the convulsive behavior was followed by full paralysis that continued well after resumption of normal lighting (Figure 1F, Video S6). This phenotype was also progressive, as young animals did not exhibit convulsions or paralysis (Figure 1F).

### Energy buffering capacity and energy levels over time in *ATP6^1^* animals

**Figure 2:**
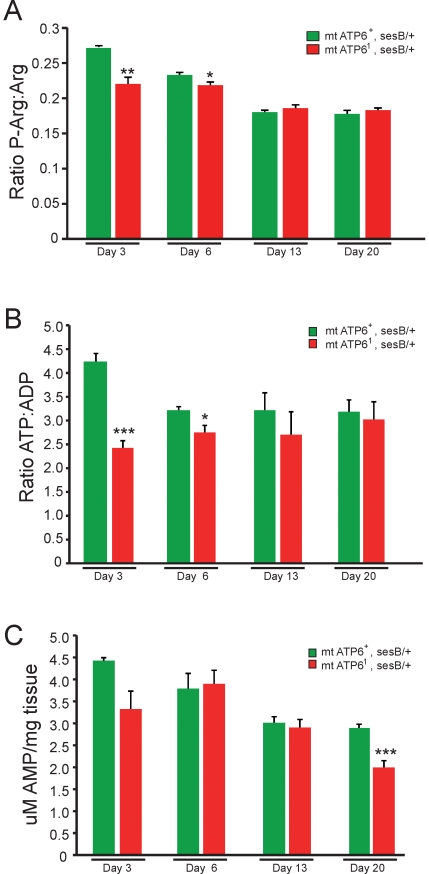
Bioenergetics of wildtype and *ATP6^1^* animals. A) P-Arg:Arg ratios exhibit impaired bioenergtics in *ATP6^1^* animals only at adult day 3 and 6 compared to age-matched wildtype. All mutant genotypes are mt *ATP6^1^, sesB^1^/+* and wildtype controls are *mt ATP6^+^, sesB/+. sesB^1^* (recessive stress sensitive B mutation) is the fly homologue to ANT (adenine nucleotide translocase) and *ATP6^1^* is maintained in this mutant background and the heteroplasmy is verified by RFLP analysis prior to experimentation (Data not shown). F1 female progeny heterozygous for *sesB^1^* are of high mutant heteroplasmy and were analyzed compared to *sesB^1^* heterozygote controls. N = 9 wildtype at each time point, N = 6 *ATP6^1^* day 3 and 13, N = 9 *ATP6^1^* day 6 and 20. Error is S.E.M. Statistical analysis is student's t-test. B) The ATP:ADP ratio analyses show a similar reduction in bioenergetics only in young mutants. N = 9 wildtype at each time point, N = 6 *ATP6^1^* day 3 and 13, N = 9 *ATP6^1^* day 6 and 20. Error is S.E.M. Statistical analysis is student's t-test. C) Comparison of uM AMP per mg of tissue between wildtype and *ATP6^1^* animals show a modest decrease in AMP in aged mutants at day 20. No significant changes are seen in total adenylate pool in *ATP6^1^* animals. N = 9 wildtype at each time point, N = 6 *ATP6^1^* day 3 and 13, N = 9 *ATP6^1^* day 6 and 20. Error is S.E.M. Statistical analysis is student's t-test.

We examined the effect altered *ATP6^1^* physiology had on animal bioenergetics. We examined phosphoarginine (P-Arg), arginine (Arg) as well as the adenylate pool using distinct HPLC protocols (Figure 2). P-Arg is the invertebrate equivalent to phosphocreatine, which buffers ATP levels and provides a reliable measure of bioenergetics [31]. Since we observe progressive pathogenesis in *ATP6^1^* animals, we analyzed the bioenergetic state over a relevant time course from asymptomatic to late-stage pathogenesis. Wildtype animals exhibit a reduction in P-Arg:Arg ratios over the first two weeks of their adult life that appear to plateau around 0.18 (Figure 2A). Surprisingly, *ATP6^1^* animals also plateau at ∼0.18 when aged, however, in the first week of their adult life P-Arg:Arg ratios are significantly reduced from that of age-matched wildtype control animals (Figure 2A).

We also examined the adenylate pool (ATP, ADP and AMP) from mutant and control animals using the same time course and the identical trend was observed (Figure 2B). A surprising decrease in the ATP:ADP ratio can be seen at days 3 and 6 (when mutants are largely aphenotypic) but no change was noted at later time points when the phenotypes are marked in severity. These data are in agreement with the data from the P-Arg:Arg assays. We also noted an age dependent decrease in AMP unique to *ATP6^1^* animals (day 20; Figure 2C). However, no change in overall total adenylate pool was noted (data not shown). These data surprisingly demonstrate that bioenergetic impairment is not likely to cause pathogenesis and suggests the importance of compensatory metabolic pathways in delaying disease pathogenesis.

### Metabolic compensation: glycolysis

**Figure 3:**
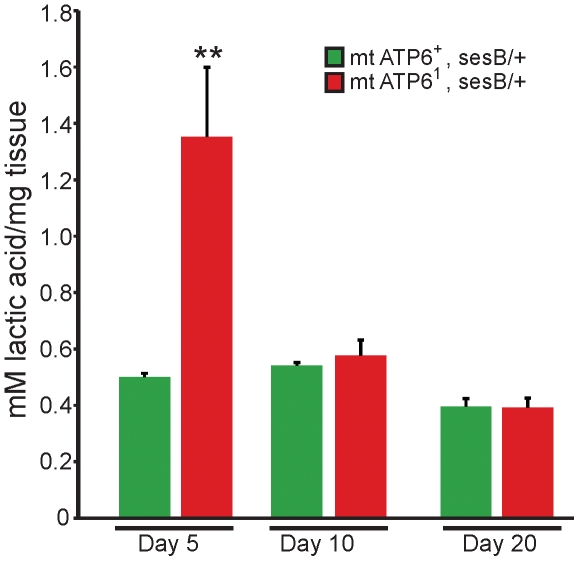
Glycolytic compensation in young *ATP6^1^* animals. A significant increase in lactic acid levels is seen in *ATP6^1^* animals on day 5 compared to wildtype animals. Steady state lactic acid is unchanged between wildtype and mutants at day 10 and 20. N = 9 wildtype at each time point, N = 9 *ATP6^1^* day 5 and 10, N = 6 *ATP6^1^* day 20. Error is S.E.M. Statistical analysis is student's t-test.

Substrate level phosphorylation occurs in glycolysis when phosphoenol pyruvate is converted to pyruvate and in the Kreb's cycle when succinyl-CoA is converted to succinate. It is thus predicted that without a functional ATP synthase (Complex V), 2 net ATP per glucose are produced through glycolysis and 1 GTP per acetyl-CoA can be produced through the Kreb's cycle. Additionally, it has been demonstrated in cell cybrid models that defects in respiratory chain complexes leads to an upregulation of glycolysis [32]. Using steady state lactic acid as a measure of glycolytic flux, a dramatic increase can be seen in young *ATP6^1^* animals (day 5) compared to age matched controls (Figure 3). Later in life (days 10 and 20) these levels have dropped to wildtype levels. These data demonstrate that increased glycolysis is an important early compensatory mechanism that is ineffective at fully abating the reduced bioenergetics observed prior to pathogenesis. These data also imply that lactic acidosis is not likely to account for the severe pathogenesis observed late in life (∼days 15–25).

### Metabolic compensation

#### Ketogenesis

**Figure 4:**
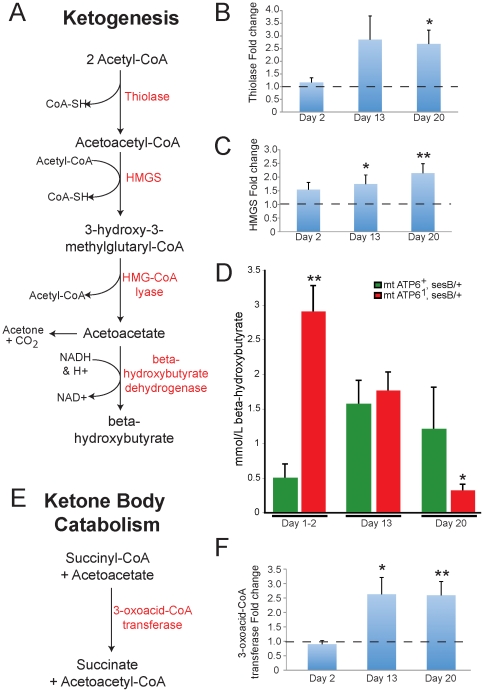
Ketogenic compensation in *ATP6^1^* animals. A) The ketogenic metabolic pathway is used to produce ketone bodies (acetoacetate, acetone and beta-hydroxybutyrate). Enzymes shown in red. B & C) Real-time quantitative RT-PCR of the enzyme thiolase and 3-hydroxy-3-methyl-glutaryl-CoA synthase (HMGS) reveal an increasing trend in *ATP6^1^* animals with age compared to wildtype. N = 12 wildtype each time point. N = 12 *ATP6^1^* each time point. Error is S.E.M. Statistical analysis is student's t-test. D) Quantitation of beta-hydroxybutyrate shows a marked increase in young animals with a decreasing trend with age compared with wildype. N = 9 wildtype day1–2, N = 5 wildtype days 13 and 20. N = 6 *ATP6^1^* day 1–2, N = 5 *ATP6^1^* days 13 and 20. Error is S.E.M. Statistical analysis is student's t-test. E) The enzyme 3-oxoacid-CoA transferase is involved in ketone body catabolism. F) Real-time quantitative RT-PCR of 3-oxoacid-CoA transferase reveals an increasing trend with age in *ATP6^1^* animals versus controls. N = 12 wildtype each time point. N = 12 *ATP6^1^* each time point. Error is S.E.M. Statistical analysis is student's t-test.

When animals are metabolically stressed, such as during starvation, they transition to utilizing fatty acids through beta-oxidation and ketogenesis. We investigated the hypothesis that *ATP6^1^* animals are utilizing ketogenesis to compensate for OXPHOS impairment. Ketogenesis is a multi-step pathway that converts acetyl-CoA to ketone bodies (acetoacetate, acetone and beta-hydroxybutyrate) that can be utilized by numerous tissues in the body, including the brain, to produce energy (Figure 4A). The expressions of two key enzymes, involved in ketogenesis, were measured over the life of *ATP6^1^* animals to determine the likelihood that this pathway is up-regulated. Both thiolase and 3-hydroxy-3-methylglutaryl-CoA synthase (HMGS) are up-regulated in *ATP6^1^* animals compared to wildtype animals beginning at day 13 (Figure 4B and 4C, respectively). Additionally, elevated beta-hydroxybutyrate was observed in *ATP6^1^* animals versus control animals early in adult life (Figure 4D). However, the ∼6-fold increase in steady state beta-hydroxybutyrate levels seen in young mutants are not maintained as *ATP6^1^* animals age and there is a reduction compared to wildtype by day 20, a time coincident with a marked worsening of *ATP6^1^* phenotypes (Figure 4D).

To understand whether this decrease in beta-hydroxybutyrate was due to the inability to maintain elevated ketone bodies or an increase in catabolism, 3-oxoacid-CoA transferase was measured. This enzyme is involved in ketone body catabolism converts of succinyl-CoA and acteoacetate (a ketone body) to succinate and acetoacetyl-CoA (Figure 4E). The expression level of this enzyme, in *ATP6^1^* animals, shows an ∼2.5-fold increase in expression compared to wildtype at days 13 and 20 (Figure 4F). These data suggest that both synthesis and catabolism are increased and that catabolism must be increased relative to synthesis to produce lower steady state beta-hydroxybutyrate levels.

### Respiration rate changes in *ATP6^1^* and wildtype animals over lifespan

During OXPHOS, oxygen is the final acceptor of electrons that are passed across the inner mitochondrial membrane at complex IV and is ultimately reduced to water. CO2 is produced during the transition from glycolysis to the Kreb's cycle where pyruvate dehydrogenase converts pyruvate to acetyl-CoA and within the Kreb's cycle at the conversion of isocitrate to alpha-ketoglutarate and the conversion of alpha-ketoglutarate to succinyl-CoA. Respiration is typically intimately coupled to mitochondrial energy production and *ATP6^1^* are severely deficient in OXPHOS, suggesting respiration would be a key parameter to understand bioenergetics and pathogenesis resulting from ATP6 impairment. To determine whether there is an age-specific change in metabolic rate, we assayed rates of CO2 production in wildtype and *ATP6^1^* flies.

**Figure 5:**
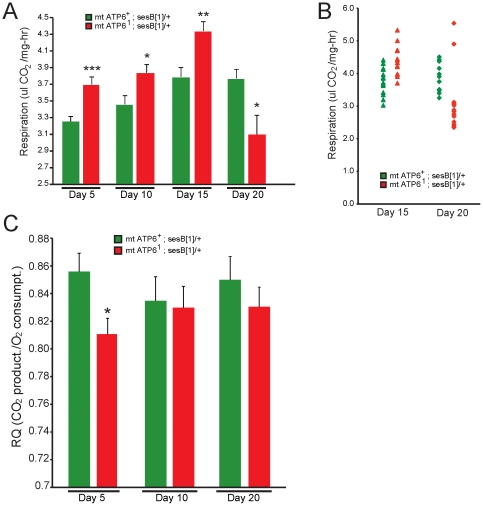
Respiration and respiratory quotient (RQ) changes over lifespan. A) Average respiration rate (ul C02/mg-hr) in wildtype (green) and *ATP6^1^* (red) animals over time. On days 5, 10 and 15 *ATP6^1^* animals have a modest but significantly higher respiration rate than wildtype animals; however, by day 20 *ATP6^1^* mutants show a significant decrease in respiration rate. N = 21−24 animals per time point per genotype. Error is S.E.M. Statistical analysis is student's t-test. B) Individual animal respiration rates at days 15 and 20. C) Respiratory quotient (RQ) of wildtype and *ATP6^1^* animals. There is a modest but significant, decrease of the RQ of *ATP6^1^* animals at day 5 compared to wildtype. N = 6−9 chambers per time point per genotype. Error is S.E.M. Statistical analysis is student's t-test.

Respiration rate was measured in individual animals at days 5, 10, 15 and 20 as the emergence of CO2 (Figure 5A and 5B). A natural trend emerged in wildtype animals, where the respiration rate increased over time plateauing between days 15–20. These data demonstrate a normal change in respiration and metabolic physiology associated with aging in our wildtype strain. Interestingly, *ATP6^1^* animals also exhibit a similar trend, however, the increase observed through day 15 dramatically reverses by day 20 (Figure 5A). By examining the single animal data the trend of increasing at day 15 and the dramatic reduction observed by day 20 for most animals can be clearly seen with only a few outliers (Figure 5B). These data demonstrate that *ATP6^1^* animals exhibit modestly elevated respiration during the aphenotypic period, and that onset of pathogenesis is associated with a striking increase in respiration followed by a dramatic drop in respiration corresponding to severe phenotypic impairment.

The dynamic changes observed in metabolic physiology and respiration over time could also be mediated through changes in mitochondrial metabolic substrate utilization. An estimate of substrate use can be obtained by comparing the ratio of CO2 produced during respiration to the amount of O2 consumed. This ratio termed the respiratory quotient (RQ) varies from approximately 0.7 for pure lipid metabolism to 1.0 for mitochondrial carbohydrate metabolism [33]. We determined the RQ for 5, 10 and 20 day old animals (Figure 5C). The RQ values for wildtype and *ATP6^1^* animals were between 0.8 and 1.0 indicative of a largely carbohydrate based metabolism. However, young *ATP6^1^* animals' exhibit a lower RQ value consistent with an increased usage of fatty acids. Overall, these respiration and RQ data suggest that *ATP6^1^* animals may be increasing their utilization of the Kreb's cycle and thus producing modestly more CO2.

### Kreb's Cycle compensation in *ATP6^1^* animals

**Figure 6:**
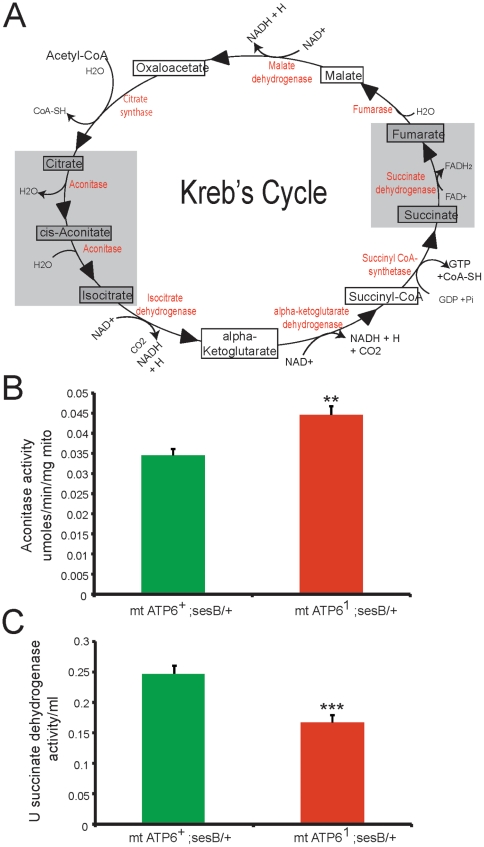
Succinate dehydrogenase and aconitase enzyme activity measurements. A) Kreb's cycle pathway; intermediates shown in boxes, enzymes shown in red. Gray boxes represent steps where enzyme activity level was measured in isolated mitochondria. B) Aconitase activity measured in wildtype (green) and *ATP6^1^* (red) mitochondria. *ATP6^1^* mitochondria have increased aconitase activity compared to wildtype. N = 15 per genotype (0–100 ug mitochondria). Error is S.E.M. Statistical analysis is student's t-test. C) Succinate dehydrogenase activity measured in wildtype (green) and *ATP6^1^* (red) mitochondria. *ATP6^1^* mitochondria have reduced succinate dehydrogenase activity compared to wildtype. N = 9 per genotype. Error is S.E.M. Statistical analysis is student's t-test.

To test the hypothesis that the Kreb's cycle activity has increased in *ATP6^1^* animals, aconitase and succinate dehydrogenase (complex II) were measured. Aconitase converts citrate to isocitrate and succinate dehydrogenase converts succinate to fumarate after GTP production in the Kreb's cycle (Figure 6A). Aconitase activity is significantly increased in *ATP6^1^* mutant mitochondria compared to wildtype (Figure 6B). However, succinate dehydrogenase activity is decreased in *ATP6^1^* mitochondria compared to wildtype (Figure 6C). These data suggest that *ATP6^1^* animals may be attempting to increase their utilization of the Kreb's cycle to maintain elevated energy levels as is seen with an increase in the aconitase step; however, later steps (succinate dehydrogenase) are unable to keep up a wildtype level of activity.

### Complex V instability and loss of Supercomplex formation in *ATP6^1^* mitochondria

**Figure 7:**
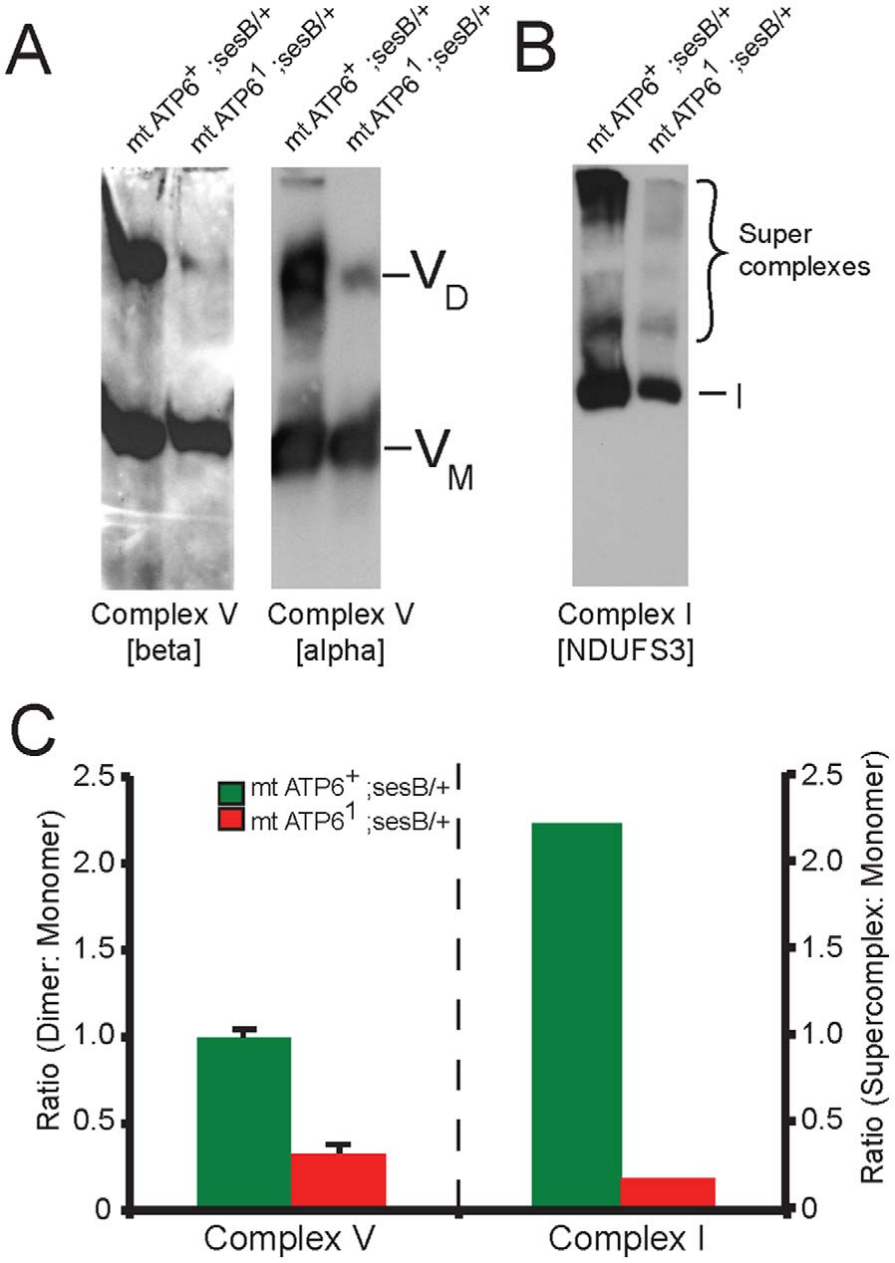
Impaired complex V dimerization and complex I supercomplex formation. A) BN-western analysis with beta and alpha subunit antibodies reveals a marked reduction in complex V dimer (VD) relative to monomer (VM) in *ATP6^1^* mitochondrial isolates compared to wild type controls. B) BN-western analysis using an antibody to complex I (NDUFS3) reveals a loss in normal supercomplexes in *ATP6^1^* mitochondria. C) Quantitation of the westerns shown in panels A and B comparing WT (green) to *ATP6^1^* (red): dimer to monomer ratio of complex V and supercomplex to monomer ratio of complex I.

TEM tomography of *ATP6^1^* mitochondria demonstrated a vesicular inner mitochondrial membrane appearance lacking the normal flattened cristae morphology seen in wildtype mitochondria [4]. Using blue-native gel electrophoresis and western blot analysis, we found impairment in complex V dimerization in *ATP6^1^* animals (Figure 7A and 7C). These data imply that mutant ATP6 protein is being expressed, yet only a small fraction of the complex V is able to dimerize. Amazingly, this mutation also leads to the disruption of complex I containing supercomplexes (Figure 7B and 7C). The changes observed in complex I supercomplexes suggest a functional connection between complex I and V, that has not previously been appreciated and may also explain the observed decrease in complex II activity in *ATP6^1^* animals.

## Discussion

Mitochondrial encephalomyopathies are devastating diseases that are difficult to diagnose and treat. Mitochondrial dysfunction results directly from mutations in the proteins involved in mitochondrial function, such as components required for OXPHOS, as is the case for archetypal mitochondrial diseases. Numerous other common disorders such as Alzheimer's disease, diabetes, cardiovascular disease, obesity and premature aging have also been associated with mitochondrial dysfunction [34], [35], [36], [37], [38], [39], [40], [41]. Understanding metabolic compensatory mechanisms that occur during chronic mitochondrial dysfunction will be needed to fully understand pathogenesis and develop effective treatments for these disorders. Utilizing an amenable intact animal model of mitochondrial encephalomyopathy has allowed for the study of disease progression and a more complete understanding of the associated pathogenesis.

The *ATP6^1^* pathogenic phenotypes are not due to bioenergetic crisis. P-Arg:Arg ratios, as well as ATP:ADP ratios, show a significant decrease compared to wildtype animals during *ATP6^1^* animals' first week of life. However, these lower levels are during a time when *ATP6^1^* animals do not exhibit signs of mitochondrial pathogenesis. When *ATP6^1^* animals begin to exhibit locomotor phenotypes as well as later in pathogenesis, energy levels are maintained at normal levels. These data demonstrate that bioenergetic crisis does not underlie pathogenesis.

The phenotypic progression in *ATP6^1^* animals includes: reduced locomotor function, sensitivity to mechanical stress, unprovoked seizure behavior, and light-induced convulsions that result in paralysis. Seizure behavior and induced convulsions demonstrates, in this invertebrate model, a neurological link between mitochondrial dysfunction and seizure activity that is also commonly seen in mitochondrial disease patients [42], [43], [44]. Conditional paralysis associated with bang- or stress-sensitivity was originally reported many decades ago and this phenotype has remained mysterious and controversial [45]. Our data demonstrate that sensory hyperstimulation alone is sufficient to cause paralysis in this bang-sensitive mutant and that this stimulation causes convulsive seizure behavior that leads to the observed paralysis.

**Figure 8:**
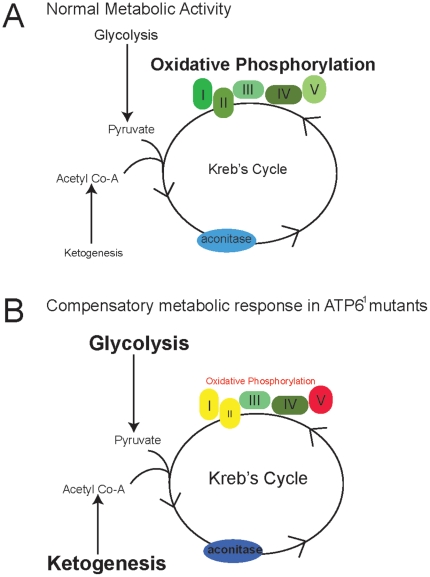
Model summarizing the dynamic metabolic changes resulting from ATP6 impairment compared with normal metabolism. A) During normal metabolic activity, oxidative phosphorylation is the major producer of ATP in the cell. Glycolysis, ketogenesis and the Kreb's cycle contribute as needed. B) During chronic ATP6 dysfunction these less utilized pathways (glycolysis and ketogenesis) are upregulated to compensate for the loss of oxidative phoshorylation. Complex V is unable to form a dimer and lacks ATP synthase capacity. Complex I supercomplexes are missing and complex II activity is down, however, there is a measurable increase in aconitase activity (an additional component of the Kreb's cycle).

*ATP6^1^* animals compensate for their reduced ability to produce ATP through OXPHOS by increasing their usage of other metabolic pathways (Figure 8). The pathogenesis seen in *ATP6^1^* animals does not appear when the activity of the ketogenic and glycolytic pathways remain elevated, suggesting their ability to effectively compensate for the OXPHOS dysfunction. It is likely that pathogenesis results from an inability to maintain these compensatory mechanisms chronically or that chronic compensation is toxic. Further studies will be needed to distinguish between these possibilities.

Respiration has previously been measured over the lifespan of several wildtype organisms resulting in conflicting reports on metabolic rate changes with age [46], [47], [48], [49], [50]. We have found wildtype animals demonstrate an increase in respiration early in life that plateaus as they age. *ATP6^1^* animals exhibit an early increase in CO2 production during the first 2 weeks of their life that is elevated relative to wildtype, however, this is followed by a drastic reduction by day 20. This suggests that these animals are trying to increase their utilization of the Kreb's cycle early in their life; however they may be unable to maintain this level of activity later in life. Additionally, *ATP6^1^* animals have a lower RQ value at day 5 and a similar value to wildtype throughout the rest of their life. This decrease in RQ value at day 5 is consistent with the observed increase in utilization of ketogenesis, a lipid-utilizing pathway.

The aberrant mitochondrial morphology seen in these *ATP6^1^* animals can be attributed to the lack of complex V dimerization. Recent work demonstrates that complex V dimer formation is necessary for the bending of the inner mitochondrial membrane giving cristae their characteristic elongated appearance and placing complex V in the proper position to utilize the highest concentration of hydrogen ions for catalysis [2], [51], [52], [53], [54], [55], [56]. The loss of efficient ATP synthase activity is either due to the inability of complex V to dimerize or due to the malformed cristae, which need to be elongated to concentrate the hydrogen ions for proper ATP synthesis. Additionally, we show that this single missense mutation disrupts the formation of supercomplexes containing complex I. This loss of supercomplex formation may have a causal relationship to the decrease in succinate dehydrogenase (complex II) activity observed, thus preventing the Kreb's cycle from functioning at an increased level.

In conclusion, we have studied the metabolic pathways within *ATP6^1^* animals and discovered a dynamic interplay between compensatory mechanisms that results when efficient production of ATP through OXPHOS is not possible. These compensatory responses allow the animal to maintain largely normal levels of ATP, demonstrating that bioenergetic crisis does not underlie pathogenesis. This normal level of ATP may be at the expense of the animals' normal activity level during the second half of their lives and thus may contribute to the observed locomotor phenotypes. Importantly, nearly every parameter examined was dynamic over the lifespan of *ATP6^1^* animals, underscoring the importance of studying such diseases as a time course in an intact animal model.

## Materials and Methods

### Fly Strains, longevity, survival, egg laying and development

All mutant genotypes were mt *ATP6^1^, sesB^1^/+,* wildtype controls were *mt ATP6^+^, sesB/+. sesB^1^* (recessive stress sensitive B mutation) is the fly homologue to ANT (adenine nucleotide translocase) and *ATP6^1^* is maintained in this mutant background and the heteroplasmy is verified by RFLP analysis prior to experimentation (Data not shown). F1 female progeny heterozygous for *sesB^1^* are of high mutant heteroplasmy and were analyzed compared to *sesB^1^* heterozygote controls, unless otherwise noted. Longevity was examined as previously published [57], [58], [59], [60], [61], [62], [63]. *Survival:* females were allowed to lay eggs in a new vial for 4 consecutive days, each day the eggs were counted and the females were moved to a new vial (adjustment made for unfertilized eggs). *Egg laying:* number of embryos laid per female was determined in 12-hour intervals over a 3-day period. *Development:* times to transition to the next stage was monitored every 12 hours at each temperature.

### Extract preparation for HPLC assays

Animals were rapidly frozen using liquid nitrogen, weighed then homogenized with an electric homogenizer in 200 µl 0.6 M perchloric acid and were then neutralized by the addition of 25 µl of 2 M potassium carbonate. Samples were centrifuged at 12,000xg for 10 minutes at 4°C. The supernatant was then filtered through a PVDF 0.45 µm spin column at 12,000xg for 5 minutes at 4°C.

### Phosphoarginine

#### Arginine ratios

HPLC protocol to measure phosphoarginine and arginine ratios was adapted from an established method [64]. Ten µl of extract was injected. HPLC conditions: Phenomenex Luna 5 µm NH2 250×4.6 mm column and 4.6×3 mm 3 µm NH2 Guard column. Flow rate of 0.6 ml/min and detection wavelength of 205 nm. A linear mobile phase consisting of 95∶5 20 mM KH2PO4 pH 2.6: Acetonitrile. Arginine standard was used in the linear range of 0.1–5 mM. Phosphoarginine standard was synthesized using arginine kinase from Homarus vulgaris longitudinal muscle similar to a published method [65]. Retention times were 3.7 and 5.3 minutes for arginine and phosphoarginine, respectively.

Adenylate Pool. HPLC protocol to measure ATP, ADP and AMP levels was adapted from an established method [66]. Twenty µl of extract was injected. HPLC conditions: Waters XBridge Shield RP18 150×4.6 mm 5 µm column and guard column. Flow rate of 0.8 ml/min, detection wavelength of 257 nm and column temperature of 30°C. A gradient mobile phase was used: time 0–6.5 minutes 0% B, 6.5–12.5 minutes 100% B, 12.5–25 minutes 0%B. Buffer A was 50 mM NH4H2PO4 pH 5.7, Buffer B was 60∶40 Acetonitrile: H2O. ATP, ADP and AMP standards were linear through the range of 100–250 µM. Approximate retention times were 4.2 minutes for ATP, 4.7 minutes for ADP and 6.2 minutes for AMP.

### Lactic acid levels

HPLC protocol to measure lactic acid levels was adapted from an established method [67]. Twenty five µl of extract was injected. HPLC conditions: Waters Atlantis dC18 150×4.6 mm 3 µm column and 4.6×20 mm 3 µm Guard column. Flow rate of 0.5 ml/min, detection wavelength of 190 nm and column temperature of 30°C. A linear mobile phase consisting of 10 mM NaH2PO4 pH 2.5. Lactic acid standard was used in the linear range of 2–10 mM with an approximate retention time of 7.6 mintues.

### Beta-hydroxybutyrate assay

Animals were cold slowed on ice before being homogenized in 10 µl PBS using a plastic pestle in a centrifuge tube. Extract was immediately read using an Optium Xceed Meter (Abbott) containing an Optium Plus β–ketone test strip. Readout was given in mmol/L.

*Quantitative RT-PCR*. RNA was prepped using Qiagen RNAeasy kit. Quantitative RT-PCR was performed as previously published [60].

### Respiration rate

Resting metabolic rates were measured on individual wildtype and *ATP6^1^* flies at 4 ages (5, 10, 15 and 20 days post-emergence, n = 21−24 animals per time point per genotype) using methods similar to those we have previously described [68], [69].

RQ measurements. To determine the relationship between CO2 production and O2 consumption, both variables were measured in wildtype and *ATP6^1^* flies at 4 ages (5, 10 and 20 days post-emergence). Groups of 5–6 flies (n = 6–9 chambers per time point per genotype) were measured using a previously described protocol [69].

### Mitochondria isolation

A continuous percoll gradient was used to isolate mitochondria from 1.5–2 g of wildtype or *ATP6^1^* adults. Protocol was adapted from an established method [70]. Between 1.5 g and 2 g of adult flies between the ages of 1–7 days were placed in 10 ml of cold buffer A (250 mM sucrose, 1 mM EDTA, 50 mM Tris-HCl pH 7.4 with protease inhibitors added before use) homogenized and centrifuged. The supernatant was centrifuged at 10,000xg for 15 minutes and was washed. The pellet was then resuspended in 1 ml buffer B (250 mM sucrose, 1 mM EGTA, 10 mM Tris-HCl pH 7.4), placed on top of a continuous percoll gradient (2.2 ml 2.5 M sucrose, 6.65 ml 100% percoll, 12.25 ml 10 mM Tris-HCl pH 7.4, 84 µl 0.25 EDTA) and centrifuged at 47,000xg for 45 minutes. The mitochondria layer was removed with a syringe and washed.

Aconitase activity. Mitosciences kit MS745 was used. Aconitase activity is measured by following the conversion of isocitrate to cis-aconitate through the increase of absorbance at 240 nm.

### Succinate dehydrogenase activity

Protocol was adapted from an established method [71]. Activity of sucinate dehydrogenase (U/min) was calculated as the change of absorbance over time and multiplied by 5.18 (due to the molar absorbance of INT-formazan).

### Blue native gel electrophoresis and western analysis

Blue Native protocol was adapted from an established method [72]. 150 µg of mitochondrial protein was loaded per well. A gradient gel was poured with 5% acrylamide:bis-acrylamide for the light component and 12% for the heavy component (plus glycerol at 11.4%), a final concentration of 50 mM bis-tris and 0.5 M 6-aminohexanoic acid (APS and temed for polymerization). Gels were run at 4°C, 50 V for 20 hours. Western analysis used the following antibodies: complex V beta subunit (Invitrogen A21351), complex V alpha subunit (Mitosciences MS507), complex I NDUFS3 (Mitosciences MS112).

### Seizure-like activity movie analysis

Flies were recorded using PAX-it version 6 software (Midwest Information Systems, Inc., Villa Park, IL) through a PAXCAM (camera) mounted on a ZEISS microscope (W.E.L. Instrument Co). Fly movements were recorded for 5 minutes then were treated with a strobe light (SHIMPO, Itasca, IL) at a frequency of 1450 flashes per minute for 20 seconds. After the strobe light treatment, videotaping of the fly movements continued for an additional 5 minutes. Video analysis was performed using iMovie (Apple, Cupertino, CA). The recovery time was measured as the time between the end of strobe light treatment and the first normal fly movement (i.e. walking forward or grooming). A two-tailed Mann-Whitney U test was used for statistical analysis at day 25 (****p<0.0001).

### Statistical analysis

In all experiments standard error is represented as standard error of the mean (S.E.M.). All analyses are student's T-test where the stars represent: **** p<0.0001, ***p<0.001, **p<0.01, *p<0.05.

## Supporting Information

Video S1 **Video analysis of wildtype before strobe light, demonstrating normal behavior.**
(MOV)

Video S2 **Video analysis of wildtype during 20 second strobe light, demonstrating that wildtype animals do not change their behavior during this sensory stressor.**
(MOV)

Video S3 **Video analysis of wildtype after strobe light, again demonstrating that wildtype animals were unaffected by the strobe light.**
(MOV)

Video S4 **Video analysis of** ***ATP6^1^*** **before strobe light, demonstrating that** ***ATP6^1^*** **animals have reduced but normal activity.**
(MOV)

Video S5 **Video analysis of** ***ATP6^1^*** **during 20 second strobe light, demonstrating the convulsive seizure behavior induced by the strobe light.**
(MOV)

Video S6 **Video analysis of** ***ATP6^1^*** **after strobe light, some** ***ATP6^1^*** **animals continue convulsive seizure behavior while others exhibit paralysis.**
(MOV)
